# RNA-Seq based phylogeny recapitulates previous phylogeny of the genus *Flaveria* (Asteraceae) with some modifications

**DOI:** 10.1186/s12862-015-0399-9

**Published:** 2015-06-18

**Authors:** Ming-Ju Amy Lyu, Udo Gowik, Steve Kelly, Sarah Covshoff, Julia Mallmann, Peter Westhoff, Julian M. Hibberd, Matt Stata, Rowan F. Sage, Haorong Lu, Xiaofeng Wei, Gane Ka-Shu Wong, Xin-Guang Zhu

**Affiliations:** CAS-MPG Partner Institute and Key Laboratory for Computational Biology, Shanghai Institutes for Biological Sciences, Shanghai, China; Institute of Plant Molecular and Developmental Biology, Heinrich-Heine-University, Dusseldorf, Germany; Department of Plant Sciences, University of Oxford, Oxford, UK; Department of Plant Sciences, University of Cambridge, Cambridge, UK; Department of Ecology and Evolutionary Biology, University of Toronto, Toronto, Canada; BGI-Shenzhen, Beishan Industrial Zone, Yantian District, Shenzhen, 518083 China; Department of Biological Sciences, University of Alberta, Edmonton, AB T6G 2E9 Canada; Department of Medicine, University of Alberta, Edmonton, AB T6G 2E1 Canada

**Keywords:** *Flaveria*, RNA-Seq, Phylogenetic tree, C_4_ photosynthesis

## Abstract

**Background:**

The genus *Flaveria* has been extensively used as a model to study the evolution of C_4_ photosynthesis as it contains C_3_ and C_4_ species as well as a number of species that exhibit intermediate types of photosynthesis. The current phylogenetic tree of the genus *Flaveria* contains 21 of the 23 known *Flaveria* species and has been previously constructed using a combination of morphological data and three non-coding DNA sequences (nuclear encoded ETS, ITS and chloroplast encoded *trnL-F*).

**Results:**

Here we developed a new strategy to update the phylogenetic tree of 16 *Flaveria* species based on RNA-Seq data. The updated phylogeny is largely congruent with the previously published tree but with some modifications. We propose that the data collection method provided in this study can be used as a generic method for phylogenetic tree reconstruction if the target species has no genomic information. We also showed that a “*F. pringlei*” genotype recently used in a number of labs may be a hybrid between *F. pringlei* (C_3_) and *F. angustifolia* (C_3_-C_4_).

**Conclusions:**

We propose that the new strategy of obtaining phylogenetic sequences outlined in this study can be used to construct robust trees in a larger number of taxa. The updated *Flaveria* phylogenetic tree also supports a hypothesis of stepwise and parallel evolution of C_4_ photosynthesis in the *Flavaria* clade.

**Electronic supplementary material:**

The online version of this article (doi:10.1186/s12862-015-0399-9) contains supplementary material, which is available to authorized users.

## Background

C_4_ photosynthesis evolved repeatedly from C_3_ photosynthesis in at least 66 different lineages of angiosperms [1, 2]. Many of these evolutionary transitions are coincident with the decline of atmospheric CO_2_ concentration in the Oligocene [1, 3, 4]. Because of the C_4_ photosynthetic pathway, C_4_ plants are able to concentrate CO_2_ into the bundle sheath cells (BSC) where RuBisCO (ribulose 1,5-bisphosphate carboxylase/oxygenase) is localized [5]. This substantially reduces the inhibitory process of photorespiration and thus increases light, water and nitrogen use efficiencies in C_4_ relative to C_3_ plants [6, 7]. Since its discovery in the 1960’s, there has been much interest in understanding how C_4_ plants evolved [8, 9]. In addition to being scientifically intriguing, a thorough understanding of the evolutionary process of C_4_ photosynthesis will also help guide efforts to engineer C_4_ traits into C_3_ plants and boost crop yield and resource use efficiencies [1, 10, 11]. In recent years, the incorporation of phylogenetic methods into C_4_ studies has greatly aided the evaluation of evolutionary hypothesis concerning when, where and how the C_4_ pathway evolved [1, 12]. Genera that include both C_3_ species and C_4_ species, as well as species with intermediate phenotypes between C_3_ and C_4_, such as *Flaveria* [13] and *Heliotropium* [14], have been of particular interest for the study of the evolution of C_4_ photosynthesis. Intermediate species have features of both C_3_ and C_4_ photosynthetic pathways [13] and often differ from C_3_ species by possessing characteristics of C_4_ plants such as enhanced C_4_ enzyme expression and activity [15, 16]. A concern has been whether the intermediate species branch in sister positions to C_3_ and C_4_ clades that would support a conclusion of shared ancestry and thus that the intermediate reflects an evolutionary transitional state [11, 17], however, resolving the phylogenetic positions of intermediates often requires a data-rich phylogeny [18].

Because it has multiple C_3_ and C_4_ species, more known C_3_-C_4_ intermediate species than any other genera, and is relatively young (past 5 to 10 million years), the genus *Flaveria* has become the main model system for the study of C_4_ evolution in the eudicots [10, 19]. For over 30 years, researches with *Flaveria* species have examined the structural, physiological, biochemical and molecular variation in their leaves and genomes [12, 15, 20–23]. Collectively, these studies led to the proposal that C_4_ characters are acquired in a step-wise manner during the evolution of C_4_ photosynthesis, and recently, the rise of C_4_ character states in *Flaveria* were proposed to follow a progressive, Mount Fuji adaptive landscape model [17]. In support of this step-wise transition, a comparative study of the localization of P-protein of glycine decarboxylase (GLDP, a key enzyme in the photorespiratory pathway) into different photosynthetic types of *Flaveria* showed that the restriction of GLDP to BSC occurs gradually in going from pure C_3_ to intermediate species and then to C_4_ species [24].

An important development in the understanding of C_4_ evolution in *Flaveria* has been the publication of a well-resolved phylogenetic tree of the genus and sister taxa [18, 25, 26]. The first phylogeny of *Flaveria* was published using morphological markers by Powell [25]. This was followed by a single gene tree by Kopriva *et al*., based on the H-protein of glycine decarboxylase [26]. While both trees were limited by relatively small data sets on which to infer relationships, they did indicate two major clades are present in *Flaveria*, with each containing C_3_-C_4_ intermediate species and C_4_ or C_4_-like species in distal positions of each clade [25, 26]. This suggests the possibility of two independent origins of the C_4_ pathway in *Flaveria*.

The most widely accepted phylogenetic tree of *Flaveria* was constructed using three non-coding DNA sequences (which comprised the nuclear ribosome internal transcribed region, external transcribed region, and the chloroplast encoded *trnL-F* spacer region) combined with a number of morphological features, such as life history, leaf surface properties and capitulescence and so on [18]. The inclusion of the morphological characters resulted in a composite tree that is widely used to infer *Flaveria* relationships in order to test hypotheses of evolutionary origin using physiological, biochemical or molecular data sets [18, 25]. However phylogenies based on non-coding regions and/or loci under selection can also potentially provide different interpretations due to varying selection pressures, demography, and selection sweeps [27, 28]. Moreover, phylogenetic trees inferred from different non-coding sequences have been shown to be incongruent with each other in numerous taxa such as the Gramineae [27]. It has also been shown that phylogenetic trees inferred from morphological data often conflict with trees inferred from molecular sequence data [18, 27, 29]. Considering the increasing interest in precisely mapping out the evolutionary steps for C_4_ photosynthesis and the importance of the *Flaveria* genus in studying C_4_ evolution, it is pertinent to re-evaluate the phylogeny of *Flaveria* using the information-rich data based on molecular sequences that has recently become available [30].

With the advent of low-cost sequencing technologies, there has been a rapid accumulation of molecular sequence data for non-model species, such as *Flaveria*. Those algorithms that utilize such sequence data to construct phylogenetic trees have been recently developed [31–33]. For example, using data matrix constructed based on assembled contigs from RNA-Seq reads, a robust phylogeny of 10 mosquito species was constructed [33]. The phylogeny of 16 lice (Insecta: Psocodea) was re-constructed by combing EST data and genomic DNA-Seq data [34], and the phylogeny of 21 species from class *Actinopterygii* were generated using different sources of data, including public genome sequences, EST, mRNA, transcriptome as well as cDNA and Unigenes [35].

In this work, we present a new method to use RNA-Seq data to generate phylogenetic trees, and then use it to reconstruct the phylogeny of *Flaveria*. First, we extracted phylogentically informative sites from RNA-Seq data by directly mapping sequence reads to coding sequences (CDS) of a fully sequenced reference species (in this case *A. thaliana*). Using this data set, we generated a phylogenetic tree of 16 *Flaveria* species, including representatives of C_3_, C_3_-C_4_ intermediate, C_4_-like and C_4_ species. The resulting tree is largely consistent with the most widely referenced *Flaveria* phylogeny [18] but with slight modifications. Using our mapping strategy we provide evidence that a “*F. pringlei*” accession used in this and many prior studies is a hybrid derived from a pure parent of *F. pringlei* (C_3_) and a *F. angustifolia* (C_3_-C_4_) parent.

## Results

### Overview of RNA-Seq samples sequenced through Illumina platform

To construct a phylogenetic tree of the genus *Flaveria*, we obtained Illumina RNA-Seq data from 17 *Flaveria* species (3 C_3_, 4 C_4_, 7 C_3_-C_4_ and 3 C_4_-like intermediate species) and 3 out-group species from the *Asteraceae* (Table 1). In total there were 37 RNA-Seq samples including 34 *Flaveria* samples and 3 samples of out-group species. The 37 RNA-Seq samples were obtained from two sources. Nineteen samples were obtained from the One Thousand Plants (1KP) Consortium (http://www.onekp.com/) which were grown in the University of Toronto and 18 samples were obtained from Heinrich-Heine University (HHU) (Table 1). On average, each *Flaveria* RNA-Seq sample from the 1KP Consortium and HHU provided around 20.0 million (from 15.6 million to 27.3 million) and 27.3 million (from 23.1 million to 43.8 million) raw reads respectively (Table 1). In summary, the 34 RNA-Seq datasets of 17 *Flaveria* species covered plants grown in greenhouses at HHU (18 datasets) and the University of Toronto (16 datasets), and included six species with both samples from juvenile leaves and mature leaves, besides, the datasets were from two different sequencing protocols, i.e. pair-end reads (University of Toronto) and single-end reads (HHU) using Illumina sequencing (Methods).

**Table 1:** RNA-Seq data and cross mapping result

Sample	PS type	# read	Average length	# mapping read	% mapping read	# target CDS	% mapping target
Pair-end RNA-seq of from Illumina, read length: 75–90 bp (from 1KP)							
*F. cronquistii-j*	a	16,809,686.00	90	7,902,191.00	47.01 %	25,692.00	72.60 %
*F. cronquistii-m*	a	15,622,832.00	90	8,114,254.00	51.94 %	25,337.00	71.60 %
*F. pringlei-j*	a	20,064,474.00	90	9,372,051.00	46.71 %	26,209.00	74.07 %
*F. pringlei-m*	a	16,219,108.00	90	9,150,289.00	56.42 %	25,308.00	71.52 %
*F. angustifolia-j*	b	16,668,010.00	90	7,983,526.00	47.90 %	25,599.00	72.34 %
*F. angustifolia-m*	b	18,085,350.00	90	9,059,885.00	50.10 %	24,877.00	70.30 %
*F. pubesens-j*	b	18,897,990.00	90	11,118,929.00	58.84 %	25,933.00	73.29 %
*F. pubesens-m*	b	20,703,102.00	90	8,502,290.00	41.07 %	25,438.00	71.89 %
*F. sonorensis-j*	b	22,424,194.00	90	11,170,838.00	49.82 %	25,959.00	73.36 %
*F. palmeri-j*	c	19,329,884.00	90	10,666,300.00	55.18 %	25,669.00	72.54 %
*F. vaginata-j*	c	18,876,338.00	90	8,830,209.00	46.78 %	25,814.00	72.95 %
*F. bidentis-j*	d	25,424,874.00	90	13,020,754.00	51.21 %	26,103.00	73.77 %
*F. bidentis-m*	d	23,089,000.00	90	12,163,057.00	52.68 %	25,582.00	72.29 %
*F. kochiana-m*	d	19,220,058.00	90	10,881,823.00	56.62 %	25,359.00	71.66 %
*F. trinervia-j*	d	23,726,482.00	90	11,295,399.00	47.61 %	26,442.00	74.72 %
*F. trinervia-m*	d	27,345,748.00	90	13,030,756.00	47.65 %	25,665.00	72.53 %
*H. autumnale*	a	25,213,280.00	90	7,916,909.00	31.40 %	26,039.00	73.59 %
*Ta. parthenium*	a	19,828,848.00	75	5,069,369.00	25.57 %	26,041.00	73.59 %
*Tr. duius*	a	23,106,402.00	90	9,485,525.00	41.05 %	26,235.00	74.14 %
Average		20,560,824.20			47.67 %		73.78 %
Single-end RNA-seq from Illumina, read length: 100 bp from (HHU).							
*F. pringlei* ^*#*^	a	38,529,805.00	90.1	20,920,082.00	54.30 %	28,605.00	80.84 %
*F. robusta* ^*#*^	a	33,113,842.00	90.1	9,625,516.00	29.07 %	29,033.00	82.05 %
*F. angustifolia* ^*#*^	b	31,408,476.00	85.1	14,328,304.00	45.62 %	28,533.00	80.63 %
*F. anomala* ^*#*^	b	31,056,596.00	91.1	15,457,407.00	49.77 %	26,676.00	75.39 %
*F. chloraefolia* ^*#*^	b	39,911,614.00	89.9	18,468,621.00	46.27 %	28,375.00	80.19 %
*F. floridana* ^*#*^	b	38,236,849.00	84.9	18,685,391.00	48.87 %	28,465.00	80.44 %
*F. pubescens* ^*#*^	b	29,940,352.00	91.4	15,965,038.00	53.32 %	28,957.00	81.83 %
*F. ramosissima* ^*#*^	b	35,283,647.00	90.4	20,060,016.00	56.85 %	29,010.00	81.98 %
*F. brownii* ^*#*^	c	43,802,834.00	91.6	20,986,495.00	47.91 %	28,180.00	79.64 %
*F. palmeri* ^*#*^	c	27,804,586.00	84	12,421,541.00	44.67 %	28,926.00	81.74 %
*F. vaginata* ^*#*^	c	35,000,281.00	84	16,077,619.00	45.94 %	28,772.00	81.31 %
*F. australasica* ^*#*^	d	25,312,995.00	84.1	10,357,274.00	40.92 %	27,387.00	77.40 %
*F. bidentis* ^*#*^	d	34,333,242.00	90.9	16,600,362.00	48.35 %	27,731.00	78.37 %
*F. trinervia* ^*#*^	d	33,540,674.00	91.2	19,511,743.00	58.17 %	29,059.00	82.12 %
Averag*e*		34,091,128.00			47.86 %		0.815
*F. bidentis* ^*#*^ *-root*		34,491,406.00	91.8	16,020,332.00	46.45 %	28,180.00	79.68 %
*F. bidentis* ^*#*^ *-shoot*		36,588,034.00	91.3	17,261,488.00	47.18 %	28,465.00	80.48 %
*F. robusta* ^*#*^ *-root*		38,514,685.00	91.8	17,220,911.00	44.71 %	28,772.00	81.35 %
*F. robusta* ^*#*^ *-shoot*		23,089,000.00	90	12,163,057.00	52.68 %	25,582.00	72.33 %
Average		20,440,345.00			47.64 %		78.46 %

Note: *Abbreviations*: F: *Flaveria*, H: *Helenium*, Ta: *Tanacetum*, Tr: *Tragopogon*, −j/m: juvenile/mature leaf sample from 1KP, ^#^: leaf sample from HHU. PS. (photosynthetic) type: a: C_3_, b: C_3_-C_4_, c: C_4_-like, d: C_4_

### Mapping reads to minimal coding sequence set of Arabidopsis thaliana

The estimated divergence time between *Flaveria* and *A. thaliana* is ~120 million years (mys) [36]. The long divergence time and hence different evolutionary histories may result in gene family expansion through duplications and thus influence correct reads mapping. In order to reduce the potential artifacts from reads cross-mapping caused by paralogs, a minimal reference coding sequences (*m-CDS*) of *A. thaliana* was used as mapping template (see Methods). The *m-CDS* contained 26,152 coding sequences (CDS) that was constructed by removing redundant paralogs. During cross-species mapping of RNA-Seq reads to *m-CDS,* we mapped reads in protein-space using BLAT and only retained mapped reads with an estimated false positive discovery rate (FDR) below 1 % (*q* = 0.00745, see Methods). All samples have similar percentages of reads mapping to the *m-CDS*: 40 %–45 % of reads from the 1KP Consortium samples can be mapped to *m-CDS* reference, and 43 % ~ 56 % of reads from HHU samples can be mapped to the reference (Table 1). Thus, around 50 % of the total number of reads from all samples was used in this study. To further estimate the accuracy of cross species mapping, we used the information from paired reads to determine the percentage of reads that mapped in concordant pairs to the same gene. On average, 68 % of all mapped reads mapped in pairs, of which 99 % mapped concordantly to the same reference, suggesting a high reliability of cross species mapping.

### Cross-species CDS sequence extrapolation

**Fig. 1:**
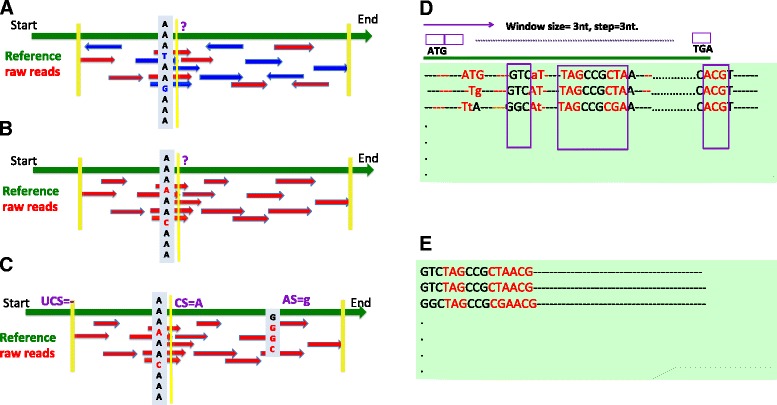
The workflow for data matrix construction. **a**–**e**: the workflow for obtaining data matrix. **a**: the coding sequence (CDS) of *A. thaliana* was used as template for mapping. RNA-Seq reads were translated into amino acid sequences and mapped to the template using BLAT in protein space; **b**: Continuously mapped reads were retained after passing minimal BLAT mapping score (see Methods), and exact read-mapped regions on the template were then extracted. **c**: UCS, CS and AS were determined by calculating the nucleotide frequency at each site based on the mapping result (see Methods); **d**: The codons were extracted from CS using sliding windows. **e**: linking retained codons for each CDS, CS data matrix was then built by concatenating retained codons from all CDS for ML method. (Abbreviations: UCS: uncovered site, CS: consensus site, AS: ambiguous site.)

Having selected the high quality mapped reads, we then used them to infer the consensus CDS sequence of each orthologous gene from *m-CDS* in each species*.* The inferred nucleotide sequence at each site was estimated based on the number of mapped reads and the frequency of occurrence of each nucleotide at each site (Fig. 1). All sites were classified into one of 3 discrete categories (see Methods): 1) consensus sites (CS), encoded in [A, T, G, C]; 2) ambiguous sites (AS), encoded in [a, t, g, c] and 3) uncovered site (UNS), encoded in gap character “-”. We then estimated the consistency of CS sites across independent leaf RNA-Seq datasets from the same species. Among six species with both juvenile and mature leaf RNA-Seq datasets from 1KP Consortium, the highest consistency was found in *F. bidentis*, with entire predicted CS sites from mature leaf samples being identical to the corresponding CS sites from juvenile leaf. 99.99 % of CS sites from *F. trinervia* were identical between juvenile and mature leaf. *F. angustifolia*, which had the lowest consistency, still showed 99.94 % of predicted CS sites being equivalent between two types of leaf libraries (Additional file 1). Finally, the CS data matrix was built by concatenating CS sites that were shared in all samples (see Methods).

### The CS based phylogenetic inference method recapitulates previously published phylogenies

**Fig. 2:**
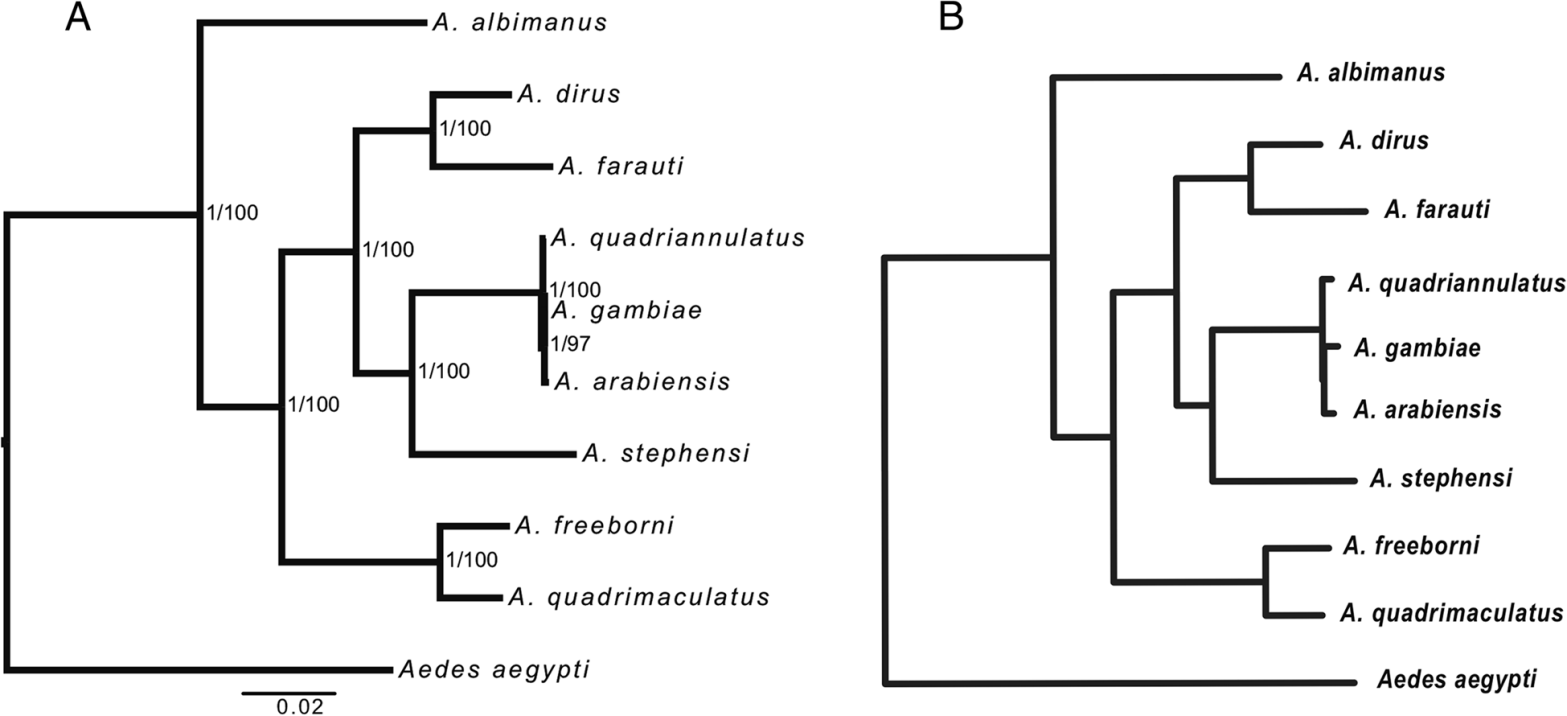
Phylogeny of ten mosquito species. **a**: phylogeny of 10 mosquito species constructed using our strategy. Both Bayesian inference (BI) tree and Maximum likelihood (ML) tree were inferred from 1,678 genes with 251,184 sites with GTR + GAMMA + I model of sequence substitution and variation. The number besides each node was posterior probability inferred from 1,000,000 generations/bootstrap score from 100 bootstrap sampling. **b**: Phylogeny of 10 mosquito species using ML method in Hittinger *et al.* (2010)

Next, we examined whether CS based phylogenetic inference method can recapitulate previously published phylogenies. Here, we applied our method to the publicly available RNA-Seq data of 10 mosquito species from the genus *Aedes* and the genus *Anopheles* in Hittinger *et al*. [33]. The reference CDS of *Aedes aegypti* were used as mapping template. This dataset was selected because the divergence time between the genus *Aedes* and the genus *Anopheles* is ~108.4 mya [36], which is comparable to the evolutionary time between *A. thaliana* and *Flaveria*. We obtained a data matrix comprising 251,184 CS spanning 1,678 genes in each species (see Methods). Both Maximum likelihood (ML) and Bayesian-inference (BI) were used to infer the phylogeny of the 10 mosquito species and the result showed that the bipartitions of the resulting ML and BI phylogenies were identical and both were the same as the published tree (Fig. 2).

### Constructed tree of individual samples of 17 Flaveria species based on m-CDS

Having demonstrated that the CS based phylogenetic inference method can recapitulate a published phylogeny in mosquito species with a well-described phylogeny, we then applied our method to RNA-Seq data from 34 individual samples of 17 *Flaveria* species. We obtained a CS data matrix comprising 315,342 CS sites from 2,183 genes (see Methods). BI tree and ML tree based on the CS data matrix had the same topology and showed the basal branching species were C_3_ and later branching species comprise the intermediates and C_4_ species (Additional file 2), which was consistent with the tree in [18]. In addition, there were also two obvious clades in our tree, only one of which contained the C_4_. However, compared with the tree in [18], our new tree showed a large shift in the position of *F. angustifolia*. The tree in [18] supported that *F. angustifolia* derived after the emergence of two clades which was supported by bootstrap score of 65 and Bayesian posterior probability of 65. However, in our tree, it is predicted to have diverged before the appearance of the two distinct clades which was supported by bootstrap score of 100 and Bayesian posterior probability of 100.

### The F. pringlei used in this study is likely a hybrid between F. pringlei and F. angustifolia

It has previously been reported that *F. pringlei* (C_3_) and *F. angustifolia* (C_3_-C_4_) can hybridize [18]. In consistent with this, they branch as sister taxa in our tree, and importantly, *F. pringlei* from both HHU and 1KP Consortium were sister taxa in the tree based on individual *Flaveria* samples (Additional file 2). Therefore, it is possible that both samples of *F. pringlei* grown by HHU and University of Toronto teams may be from hybrid seeds of true *F. pringlei* and *F. angustifolia*. To assess this possibility, we defined 8126 C_3_ and C_3_-C_4_ marker sites from 3018 genes based on the pooled leaf samples (see Methods). Out of these 8126 sites, *F. pringlei* showed 9.04 % being C_3_ marker and 20.20 % being C_3_-C_4_ marker, and 38 % being hybrid site dominant by either C_3_ marker (19.06 %) or C_3_-C_4_ marker (19.00 %) (Table 2), indicating *F. pringlei* expressed genes both from C_3_ and C_3_-C_4_ species. In contrast, in *F. angustifolia*, 85.17 % of sites were C_3_-C_4_ marker, which suggested that *F. angustifolia* belong to the C_3_-C_4_ species (Table 2). Similarly, by only analyzing *F. pringlei* sample from HHU, we found 70 % of marker sites have mapped reads being C_3_ and C_3_-C_4_ marker sites with a ratio close to be 1:1 (Additional file 3). Thus, our result suggested a high possibility of *F. pringlei* used by HHU was derived from a hybrid of true *F. pringlei* and *F. angustifolia*. Therefore, we termed *F. pringlei* in this study as *F. pri × F. ang.* To eliminate any potential influence on phylogeny construction caused by *F. pri × F. ang*, we reconstructed phylogenetic tree without the *F. pri × F. ang* samples.

**Table 2:** The percentage of sites with a C_3_ origin, or C_3_-C_4_ origin in *F. pri × F. ang, F. angustifolia* and *F. sonorensis*

	*Pulling F. pri* × *F. ang* ^a^		*F. angustifolia* (C_3_-C_4_)		*F. sonorensis* (C_3_-C_4_)	
Category	# sites	Proportion (%)	# sites	Proportion	# sites	Proportion (%)
Expressed from C_3_ allele	731	9.04	764	9.76	0	0
Expressed from C_3_ -C_4_ allele	1633	20.2	6668	85.17	6609	97.62
Expressed from both alleles	3075	38.03	18	0.23	0	0
Uncertain	2573	31.83	305	4.84	161	3.38

^a^pulled RNA-Seq date sets from HHU and 1KP to interpret the *Pulling F. pri × F. ang*

### Constructed tree of 16 Flaveria species based on m-CDS

**Fig. 3:**
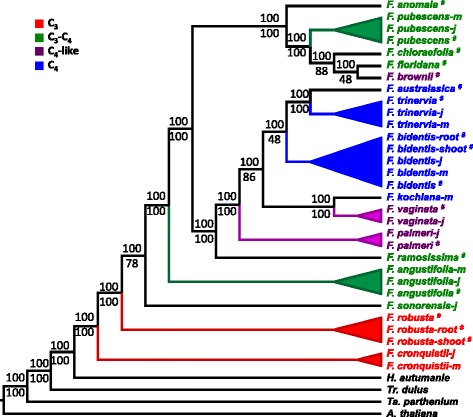
Phylogenetic tree of individual *Flaveria* samples based on *m-CDS.* To remove the effect of *F. pri × F. ang* on phylogenetic relationships among other species, the phylogenetic tree was constructed without *F. pri × F. ang*. The *m-CDS* of *A. thaliana* was used as mapping reference to construct consensus sequence (CS) matrix according to Fig. 1. A CS matrix with 343,590 sites from 2,190 genes was used to infer phylogenetic relationships based on both Bayesian inference (BI) and Maximum likelihood (ML) using GTR + GAMMA + I model of sequence substitution and variation. BI tree and ML tree showed consistent topology. The numbers besides each node were posterior probability inferred from 1000,000 generations (up) and bootstrap score (down) from 500 bootstrap sampling. (^#^/shoot^#^/root^#^/: leaf/shoot/root sample from HHU, j/m: juvenile/mature leaf sample from 1KP. *m-CDS*: reference contains the longest gene for each paralog family)

Based on the 31 RNA-Seq samples from 16 *Flaveria* species, we obtained a CS matrix comprising 343,590 CS sites from 2,190 genes (see Methods). Both the BI method and ML method were applied to infer the phylogeny based on CS matrix and the two methods generated exactly the same tree topology (Fig. 3). The tree excluding *F. pri* × *F. ang* was largely congruent with the tree including *F. pri × F. ang* (Additional file 2) but presented two alternative branching possibilities: the taxon of *F. sonorensis* (C_3_-C_4_) was exchanged with the taxon of *F. angustifolia* (C_3_-C_4_), and the taxon of *F. robusta* (C_3_) was exchanged with the taxon of *F. cronquistii* (C_3_) (Fig. 3). The resulting topology was consistent with the topology in Mckown’s tree [18], where *F. cronquistii* was the basal-branching taxon in *Flaveria*, and *F. sonorensis* emerged earlier than *F. angustifolia*. Therefore, we suggested a “dragging-to-root” effect on the position of *F. angustifolia* was caused by *F. pri* × *F. ang* in the tree containing *F. pri* × *F. ang* (Additional file 2). Notably, individual samples from different libraries of a species, such as samples from different tissues, different development stages, and different labs, were placed as the closet sister taxa (Fig. 3), suggesting that our method can be applied for RNA-Seq of plant samples from different sources or different sequencing protocols.

**Fig. 4:**
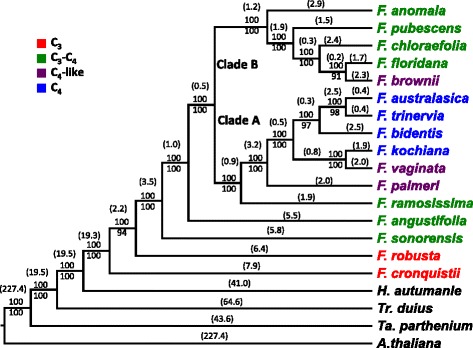
Phylogenetic tree of 16 *Flaveria* species using *m-CDS.* Pooled RNA-Seq reads of 16 *Flaveria* species were mapped to *m*-*CDS* of *A. thaliana*, consensus sequence matric was then built according to method shown in Fig. 1. Both Bayesian inference (BI) tree and Maximum likelihood (ML) tree were inferred from 2, 462 genes with 539,391 sites with GTR + GAMMA + I model of sequence substitution and variation. The numbers besides each node were posterior probability (up) inferred from 1000,000 generations and bootstrap score (down) from 500 bootstrap sampling. The numbers in brackets were relative branch length estimated from Bayesian. (*m-CDS*: reference contains the longest gene for each paralog family)

We found that some of the branches in the tree of Fig. 3 had low ML scores, e.g. the branch containing *F. brownii* and *F. floridana* (ML = 48), and the branch containing *F. bidentis*, *F. trinervia* and *F. australasica* (ML = 48). Given that trees inferred from the CS data matrix are independent of developmental stages, tissues, RNA-sequencing protocols, and growth conditions, we pooled all CS sites for the same species to increase the quantity of input data for phylogenetic tree construction. This pooling can potentially help resolve phylogenetic relationships for taxa of recent emergence. The pooled dataset comprised 20 species, including 16 *Flaveria* species and 3 out-group species together with *A. thaliana*, and the corresponding CS data matrix comprised 539,391 CS sites from 2,462 genes. As before, both ML and BI methods were used to infer the phylogeny using the CS data matrix (see Methods). The two approaches yielded identical tree topologies (Fig. 4). Importantly, the tree based on pooled samples (Fig. 4) was the same as the tree based on individual samples (Fig. 3). However, the bootstrap scores of this pooled-sample tree were on average higher than that of the tree based on individual samples, especially at the nodes that were supported by low bootstrap scores. For example, the branch containing *F. floridana* and *F. brownii* was supported by bootstrap score of 48 in the individual-sample tree, but supported by a 91 bootstrap value in the pooled-sample tree.

It has been demonstrated previously that evolutionary rates may differ between single-copy and duplicated genes [37]. To determine whether this influenced our phylogeny, we created the singleton reference CDS (*s-CDS*) dataset by removing all genes that have duplicates in *A. thaliana* from the *m*-*CDS* dataset (see Methods). The CS data matrix based on *s-CDS* containing 311,901 CS from 1,706 genes was then used to infer a phylogenetic tree of 16 *Flaveria* species by applying the same methods mentioned above (see Methods). The results showed that, the topology of the phylogenetic tree of 16 *Flaveria* species based on *s-CDS* (Additional file 4) was identical to that based on *m-CDS* (Fig. 4). But the tree based on *s-CDS* was supported by lower bootstrap scores, especially for those nodes that were supported by low bootstrap score in *m-CDS*. Our results indicate that multiple paralogs do not have a major effect on phylogeny outcomes based on RNA-Seq data.

It has been repeatedly demonstrated that 3^rd^ codon positions have different rates of evolution from those of the 1^st^ and 2^nd^ codon positions [38, 39]. To estimate the effect of different codon position on the phylogenetic tree, we constructed separate phylogenetic trees using three codon positions independently (see Methods). Results showed that the trees based on the three independent codon positions and the entire codons were largely congruent with each other in most taxa; the major difference was observed in the positions of *F. bidentis* and *F. angustifolia*. Specifically, compared with trees based on the 2nd (Additional file 5 B) and 3^rd^ codon positions (Additional file 5 C) as well as the entire codons (Fig. 4), the tree based on the 1^st^ codon position (Additional file 5 A) placed *F. bidentis* at the base of *F. kochiana* and *F. vaginata* but with a low bootstrap score of 52. The tree based on the 2^nd^ codon position, however, placed *F. angustifolia* at the base of clade A with a low bootstrap score of 74. Importantly, the topology of the phylogenetic tree inferred from the 3^rd^ codon position (Additional file 5 C) was identical to that based on entire codons (Fig. 4). This indicated that for the genus *Flaveria*, the strongest phylogenetic signal is derived from the 3rd codon position. We postulate that the short divergent time among *Flaveria* species (~5 mys) make the third codon position, which is most variable, a preferred choice in resolving the phylogenetic relationships according to [40]. This finding is consistent with previous reports that the 3rd position of codons contains the stronger phylogenetic signal [38, 39]. In this regard, it is also worth mentioning that some reports [41, 42] suggested that the 3^rd^ position of codons can also potentially bias the construction of phylogenetic trees.

## Discussion

### A new approach to construct phylogenetic trees using RNA-Seq data

Phylogenetic analyses are essential for interpreting species relationships and evolutionary transitions within lineages, in particular, the origin of complex traits such as C_4_ photosynthesis. The advances of the next generation sequencing technologies are rapidly decreasing the cost of both transcritpome and whole genome sequencing. In this study, we exploited a novel method to use transcriptome data for phylogenetic inference. Compared to genome sequencing, RNA-Seq is much cheaper and is enriched in the genetic sequences that commonly form the basis of phylogenetic analysis [43]. Moreover, RNA-Seq is biased towards highly expressed genes, which are likely to have housekeeping and energy metabolism functions [44] and thus be conserved across species [33]. Therefore, RNA-Seq has been proposed as rich data in constructing construct phylogenetic trees to study evolutionary questions for living species [33].

In prior studies that have used RNA-seq to infer phylogenetic trees, a common first step has been to assemble short reads to generate sequence contigs, and then to map these contigs onto reference transcripts [33–35]. We used an alternative approach where we directly mapped RNA-Seq reads to the reference coding sequences (CDS) of the model species *Arabidopsis thaliana (A. thaliana)*. This new method has a number of advantages: (1) it directly uses RNA-Seq reads for multiple sequence alignments in a single step and thus is suitable for scaling to a large number of species, (2) it bypasses the process of assembling short reads into contigs, thus saving computational resources and time, (3) it avoids potential errors caused by assembly, for example, misassembly or gene fusion, and (4) it avoids generating multiple sequence alignments and the potential errors introduced by this step. Although UTR and intronic regions are very useful in studying evolution, our method excludes such information because they are less conserved across species and therefore have less chance to be retained in data matrix after mapping short reads to CDS. Our method enables us to detect whether a sample is from a plant formed by hybridization of two other species, which is hard to assess using morphological data or a limited number of gene loci. This is crucial because samples from hybrid species may substantially influence the accuracy of a phylogeny. One drawback of our method is that ORFs may not be the same in *Flaveria* and in *A. thaliana*. To overcome this issue, we discarded genes with less than 10 % of the CDS region being covered by CS sites.

Though RNA-Seq based approaches are shown to be effective, for now, PCR-based and EST-based methods are still preferred initial methods for resolving phylogenetic relationships, due to their relative ease and cost. RNA-Seq methods can serve as valuable alternatives when the phylogenetic relationship cannot be solved using traditional methods due to inadequate informative sites or short divergence time between species, or when it is hard to design PCR probes for species with little genomic information. Furthermore, our CS based phylogenetic inference approach can use samples from different tissues, living conditions, developmental stages, and sequencing protocols (Fig. 3). One potential caveat is that alternative splicing specific to some tissues may result in a different CS data matrix. Our results indicate that phylogenetic relationships of 16 *Flaveria* species were equivalent between the tree based on samples from different tissues (Fig. 3) and the tree derived from samples from only leaves (Fig. 4). Therefore, the effect of alternative splicing has relatively minor impacts on the construction of the phylogenetic tree using our method.

Multiple paralogs are a potential source of error in using RNA-Seq data to generate phylogenetic tree during the process of aligning reads to a reference. In this study, we devised strategies to avoid ambiguous results caused by paralogs. Specifically, we used two different references for reads mapping. The first reference used the longest gene for each paralog family (*m*-*CDS*) in *A. thaliana*, and a separate reference used genes that have no paralogs in *A. thaliana* (*s*-*CDS*). Using *s-CDS* references effectively remove cross mapping problems although at a cost of reduced numbers of informative sites. The procedure of consensus sequence (CS) construction further eliminated the influence of cross mapping reads because we accepted one site being a CS only if this site exists in at least 10 reads, of which 80 % contains the same nucleotide. As a result, reads from paralogs, especially those with large sequence differences from the ancestor genes, should not contribute to the CS. The equivalent topology between the phylogenetic tree based on *m-CDS* and that based on *s-CDS* indicates that multiple paralogs have no effect on the topology RNA-Seq based phylogenies in the *Flaveria* case.

### An updated phylogeny of the genus Flaveria

The updated tree (Fig. 4) from this study is largely consistent with the previously published tree [18]. Our new tree also shows two distinct clades in *Flaveria* (termed clades A and B by McKown *et al*. 2005 [18]). Our tree also indicates C_3_ species branch in a basal position to intermediate and C_4_ species, thus supporting hypotheses that C_3_ photosynthesis is the ancestral condition in *Flaveria* [18]. One difference between the RNS-Seq tree and the tree in [18] is the position of *F. angustifolia*. In the previous published tree [18]*, F. angustifolia* is placed at the root of clade B with a bootstrap score of 65 (ML = 65) and Bayesian posterior probability of 65 (BI = 65). However, in our trees, *F. angustifolia* resides in the root position for both clade A and clade B (ML = 100, BI = 100), and this modification is consistent with the same number of layers (8 layers) of leaf ground tissue observed in*F. angustifolia* and two C_3_ species: *F. cronquistii* and *F. robusta* [12]. Another difference is the place of *F. chloraefolia* in clade B, which was placed near the root of clade B branching above *F. anomala* (ML = 56, BI < 50) in [18], however, the branch of *F. chloraefolia* was exchanged with the branch of *F. pubescens* (ML = 100, BI = 100) in our tree. It is interesting to note that many of the ML bootstrap scores in the currently accepted tree for clade B are less than 80 in [18]. In contrast, in the updated tree, nodes in clade B have more reliable ML scores, with the lowest ML score being 91 (Fig. 4). Our tree agrees with [18] in the placement of the *F. ramosissima* branch in clade A, however, the branches containing *F. kochiana* and *F. vaginanta* were exchanged with the branch containing *F. australasica* and *F. trinervia*.

The main reason for these differences between the RNA-seq tree (Fig. 4) and the McKpwon *et al*. tree [18] is likely due to different number of informative sites available for two methods. The informative sites used in [18] may be insufficient, which resulted in lower supports based on ML or BI at some nodes, especially for nodes in clade B. As a result, the phylogenetic positions of *F. brownii*, *F. linearis* (A–D) and *F. pubescens*, *F. oppositifolia*, *F. floridana* were not resolved because of low ML and BI supports.

#### Implications for C_4_ evolution and engineering efforts based on the updated *Flaveria* phylogenetic tree

The phylogenetic tree of the 16 *Flaveria* species suggested several features of C_4_ evolution (Fig. 4). First, clade A of the tree strongly supports hypotheses of step-wise evolution from C_3_ towards C_4_, which has been proposed based on morphological traits and physiological traits [16, 26, 45]. The progress from C_3_ to C_4_ photosynthesis in this clade started with a C_3_-C_4_ intermediate photosynthesis type in *F. ramosissima* and then to a C_4_-like photosynthesis type in *F. palmeri* [18]. Secondly, we found two parallel C_4_ sub-clades: the sub-clade containing *F. kochiana* and *F. vaginata* and the sub-clade containing *F. bidentis*, *F. trinervia*, and *F. australasica*. This repositioning presented two equally possible scenarios 1) that there was a single origin of C_4_ photosynthesis with a reacquisition of weak C_3_ activity in mesophyll cells in *F. vaginata* [16], or 2) that C_4_ has arisen twice within this clade. Both hypotheses invoked two transitions and thus are equally likely [18]. Thirdly, clade B contained only intermediate species, i.e. 5 C_3_-C_4_ species and one C_4_-like species, *F. brownii*, which shows comparable C_4_-like leaf anatomy and physiological traits as the *F. palmeri* [18, 46] in the clade A, suggesting that C_4_ traits can be recruited from multiple trajectories [47]. Such multiple trajectories towards C_4_ photosynthesis have also been previously proposed from phenotypic landscape inference [10, 19].

#### Cross fertilization in the genus *Flaveria*

Our results suggested that the *F. pringlei* (termed as *F. pri* × *F. ang* in this study) from both HHU and University of Toronto are hybrids from pure parents of a *F. pringlei* and a *F. angustifolia* (Table 2, Additional file 2). This could have happened either naturally in the field, or in cultivation in a greenhouse environment [25]. In addition to *F. pringlei* and *F. angustifolia*, many other *Flaveria* species are able to cross-fertilize, as have been summarized in Powell [25]. Therefore, it is important to check whether *Flaveria* samples are indeed pure species during experiments. Considering that none of the *Flaveria* genomes has been sequenced so far, comparing RNA-Seq reads with marker sites of defined photosynthetic types coupled with constructing a phylogeny using CS based phylogenetic inference approach provide a strategy to distinguish the hybridized or mixed samples from pure samples.

## Conclusions

In this study, we developed a new procedure to obtain abundant phylogenetic data for generating phylogenetic trees in non-model species. Using this approach, we constructed a robust phylogeny of 16 *Flaveria* species, which were largely congruent with previous public trees although the positions of some species were modified. These modifications are supported with high branch-supports. We showed that samples of *F. pringlei* (termed as *F. pri* × *F. ang*) used in a number of labs are hybrids of original *F. pringlei* (C_3_) and *F. angustifolia* (C_3_-C_4_). We propose that the new strategy on obtaining phylogenetic informative sequence from this study can be used to study phylogeny for a larger number of taxa.

## Methods

### Sample preparation and high throughput sequencing

*Flaveria* samples used in this study were from two sources. 16 leaf samples of 11 *Flaveria* species were grown in a greenhouse at University of Toronto and sequenced from One Thousand Plants (1KP) Consortium (https://sites.google.com/a/ualberta.ca/onekp/). Growth condition and plant treatment were described in detail in [48]. Leaves of two to four plants were sampled to obtain 0.1 to 1.0 g of tissue, with samples being flash frozen in liquid nitrogen and stored at −80 °C until RNA extraction. For mature leaves, the newest fully expanded leaf was chosen. For juvenile leaves, the leaves most recently starting to expand from the main stem were chosen (0.1 to 0.5 mm depending on species). Samples were taken during June and July, during long daylengths of high light, and between 9 am and 1 pm. RNA was extracted using protocol 12 of [48]. Library construction was performed as an in-house service by BGI-Shenzhen using 20 μg total RNA and the standard Illumina protocol. The second source of RNA-Seq samples was from 14 *Flaveria* species, including 14 leaf samples, two root samples and two shoot samples. Plant were grown in a greenhouse at Heinrich-Heine University (HHU) in 17-cm pots of soil (C-400 with Cocopor [Stender Erden, Schermbeck Germany] and fertilized with 3 g/L Osmocote exact standard 3 to 4 M [Scotts]). The plants received additional lighting to provide photoperiods of 16 h per day. The second and fourth visible leaves below the apex were harvested at noon and immediately frozen in liquid nitrogen and stored at −80 °C until RNA extraction. Total RNA was isolated from the second and fourth leaves according to [49]. The remaining DNA was digested with DNAse for 15 min followed by phenol and chloroform extraction and precipitation. The RNA quality was tested on a DNA chip with the Agilent 2100 bioanalyzer. 1 μg of total RNA was used for cDNA library generation with the TruSeq™ RNA Sample Preparation Kit (Illumina Lnc., San Diego, USA). Clusters were generated with the TruSeq SR Cluster Kit v2 according to the Reagent Preparation Guide with the Illumina cBot device. The RNA sequencing was performed with the Illumina platform. The RNA-Seq data of the 3 out-group species (*Tragopogon duius, Helenium autumnale*, and *Tanacetum parthenium*) were from 1KP Consortium. Their RNA isolation, library preparation and sequencing procedures were summarized in Johnson *et al.* [48], and the collection information is available at (https://sites.google.com/a/ualberta.ca/onekp/).

### Build minimal coding reference set and singleton coding reference set

We used coding sequences (CDS) of *Arabidopsis thaliana* (*A. thaliana*) as reference for reads mapping because *A. thaliana* had the most comprehensive genome annotations for dicots. We used the CDS annotation from TAIR10 (http://www.arabidopsis.org/), which contained 35,368 references. To exclude potential artifacts from reads across-mapping between paralogs [30, 50], two reference CDS sets described below from *A. thaliana* were generated and used as templates for read mapping.

The first reference set we used was a minimal reference coding sequences (*m-CDS*) set. In brief, the *m-CDS* set was built by cataloging paralogs into paralog groups and further retaining the longest gene for each paralog group. In order to remove all possible paralogs in the TAIR10 dataset (http://www.arabidopsis.org/), CDS from TAIR 10 were blasted in an “all against all” manner using the blastp program version 2.2.28 (blastall –p blastp) [51]. To choose a proper E-value cutoff for determining paralogs from blastp result, we exploited a set of known paralogs, which were predicted based on TAIR 9 dataset (http://www.arabidopsis.org/). The E-value of the upper 95 % percentile of known paralogs was used as the cutoff to catalog possible paralogs into paralog groups from TAIR 10. Finally, only the longest gene from each paralog group was retained.

More stringently, considering genes with paralog (duplicates) may have different evolutionary rate compared with the genes without duplication (singleton) [37], a singleton reference coding sequences (*s-CDS*) was generated as the second reference set, which was composed of singleton genes only.

### Read mapping and derived nucleotide sequence extrapolation

RNA-Seq reads were mapped to *m-CDS* of *A. thaliana* using BLAT in protein space with the following parameters [-t = dnax -q = dnax -repMatch = 100 -trimT] [52]. Then we defined the BLAT score cutoff for mapped reads based on the mapping false discovery rate (FDR). To estimate a FDR of mapping, we generated shuffled *m-CDS* by shuffling *m-CDS* by preserving hexamer frequency using Ushuffle (k =6) [53]. FDR was estimated to be the ratio of the number of reads mapped to shuffled *m-CDS* to the number of reads mapped to *m-CDS*. Then at FDR less than 1 %, reads passed BLAT score cutoff (49 for 1KP samples and 39 for *Tanacetum pathenium*, and 54 from HHU samples) were considered as mapped reads.

Mapped reads were used to extrapolate the derived nucleotide sequence at each site for each gene in each sample (Fig. 1). Specifically, for each gene with aligned RNA-Seq reads, we calculated the frequency of each nucleotide [A, T, G, C] at each site. The derived nucleotide sequence at each site was extrapolated as follows: if a site had greater or equal to 10 mapped reads and no less than 80 % of mapped reads reported the same nucleotide at this site e.g. “A”, then the site was assigned to be a consensus site (CS) with confident nucleotide, i.e. “A”. Otherwise, the site had no consensus nucleotide. The latter case can be further divided into two categories. If there was no read mapped to this site, the site was assigned to be an uncover site (UNS) and a symbol “-” was assigned to the site, and if there were reads mapped to this site, but either less than 10 reads mapped to or less than 80 % of mapped reads reported the same nucleotide, the site was assigned as ambiguous site (AS) and lower letter of nucleotide reported by the nucleotide with the highest number, e.g. “a”. Here we used *F. bidentis* as an example to illustrate this scenario. 10 reads were mapped to the second nucleotide site of the reference transcript AT5G54320.1 and eight of these reads reported “A”, therefore, the second site of this transcript was assigned as “A”. Site 20 was assigned as “-” because no read was mapped to this site. The 30th site was assigned as “a” because 12 reads were mapped to this site, among them 8 reads mapped with “A” and 4 reads mapped with “T”. Finally, only genes with CS sites covered at least 10 % of their CDS lengths were retained in this study. To build CS data matrix, we first kept the CS sites only if they were CS sites across all samples, and then these CS were linked codon-by-codon for each CDS, which generated the CS data matrix. It should be noted that as all inferred CDS were constrained to the same *A. thaliana* reference sequence, therefore, there was no need for construction of multiple sequence alignments following concatenated sequence.

### Investigating consistency of CS of juvenile and mature leaf libraries from the same species

To examine the accuracy of our CS extrapolation method, a proportion of identical CS sites between juvenile leaf and mature leaf of the same species were calculated for each of six species that their RNA-Seq samples were available for both developmental stages. For each transcript, the length of CS was first calculated from both juvenile leaf and mature leaf. The identical CS sites between the two libraries were then retained using in house Perl script, and the number of identical CS between juvenile leaf and mature leaf was plotted against the number of CS of mature leaves. The mean percentage was calculated for all transcripts in each species, shown at the bottom-right corner inside each panel figure (Additional file 1).

### Reconstructing phylogeny of ten mosquito species based on CS extrapolation method

RNA-Seq data of ten mosquito species were retrieved from National Center for Biotechnology Information short read archive, with accession no. SRR031793 and SRR031794 for *Anopheles stephensi*, SRR031789 to SRR031792 for *Anopheles quadrimaculatus*, SRR031787 and SRR031788 for *Anopheles freeborni*, SRR031680 to SRR031682 for *Anopheles arabiensis*, SRR031706 and SRR031707 for *Anopheles farauti*, SRR031705 and SRR031691 for *Anopheles dirus*, SRR031667 and SRR031668 for *Anopheles albimanus*, SRR031659 to SRR031662 for *Aedes aegypti*, and SRR031663 to SRR031666 for *Anopheles gambiae*. Assembled cDNA reference of *Aedes aegypti* was downloaded from https://www.vectorbase.org/. CDS of *Aedes aegypti* was predicted based on assembled cDNA using Orfpredictor [54], which resulted in 18,469 predicted CDS from a total of 18,769 cDNA. RNA-Seq reads of ten mosquitoes were mapped to these 18,469 CDS reference of *Aedes adgypti* using BLAT with the parameters [-t = dnax -q = dnax -repMatch = 10000 -stepSize = 5 -minScore = 18 -trimT] [52]. After constructing multiple sequence alignments using our method mentioned above, we then used both Maximum likelihood (ML) implemented in the RAxML package V. 2.2.3 [55] and Bayesian-inference (BI) from MrBayes 3.2.1 version [56] to infer the phylogeny of the ten mosquito species. In both cases, we used the GTR model of sequence evolution assuming a mutation rate following Gamma distribution with an estimate of proportion of invariant sites. Posterior probabilities in BI trees were calculated by running 1,000,000 generations and the first 2000 generations were used as burn-in. To construct the ML tree, genes were concatenated into a super gene in every species. The ML tree was inferred using the bootstrap parameter −p 1234 –b 1234 [55]. Bootstrap scores of ML tree were calculated by running 100 bootstrap samplings.

### CS-based phylogeny construction of 17 Flaveria species

The CS-based phylogenetic data matrix was built using codons for which all their nucleotides were CS in all aligned samples. Data matrix based on individual RNA-Seq library of *Flaveria* species was constructed from *m*-*CDS* (Additional file 2). We used PartitionFinder (V1.1.1) [57] to estimate the proper model for inferring phylogeny based on obtained CS matrixes. The result showed GTR + GAMMA + I model (a General Time Reversible nucleotide substitution model with assumption that variations in sites follow gamma distribution and with a portion of invariant sites in a sequence) best fitted our datasets. This model was then used to infer phylogeny for each data matrix. Both ML and BI methods were used for phylogenetic tree construction. Posterior probabilities in BI trees were calculated by running 1000,000 generations and the first 2000 generations was used as burn-in. To construct ML tree, genes were concatenated into a super gene in every sample. The ML tree was inferred using bootstrap parameter: −p 1234 –b 1234 [55]. Bootstrap scores of ML trees were calculated by running 500 bootstrap samplings. The phylogenetic trees constructed using ML and BI methods were displayed using FigureTree (http://tree.bio.ed.ac.uk/software/Figuretree/).

### Accessing hybrid results in Flaveria

To investigate the possibility of the *F. pringlei* sample used in this study being a hybrid of true *F. pringlei* and *F. angustifolia*, we tested whether the *F. pringlei* sample in the present had 1:1 maternal and paternal alleles, in another word, we calculated the ratio between the number of sequences being C_3_ maker sequence and the number of sequences being C_3_-C_4_ marker sequence in *F. pringlei*. Here, C_3_ marker sequences were defined as the sequences that all C_3_ species were consistent (without considering *F. pringlei*), and C_3_-C_4_ marker sequences were defined as those sequences that all C_3_-C_4_ species were consistent (without considering *F. angustifolia*). For example, at site 872 of reference AT1G01710.1, C_3_ marker is C, and C_3_-C_4_ marker is T. In *F. pringlei*, at this site, 58 % of mapping reads showed C and 42 % of mapping reads showed T. Therefore, *F. pringlei* expressed genes showing both C_3_ and C_3_-C_4_ markers at this site. In this study, we classified sites as hybrid sites if they expressed both C_3_ and C_3_-C_4_ genes, with both being supported by no less than 40 % of mapping reads. In contrast, if a site had no less than 90 % of mapping reads reported be either a C_3_ or a C_3_-C_4_ marker, then this site was classified to be either a C_3_ or a C_3_-C_4_ site. The third category of sites was that mapping reads showed neither C_3_ nor C_3_-C_4_ marker, which was then termed as a *F. pringlei* specific site. Based on this method, a large proportion of mixed sites observed in *F. pringlei* indicated a high probability that *F. pringlei* was from hybrid of pure *F. pringlei* and *F. angustifolia*. As a control, the number of sites belonging to each of these three categories in other two species, *F. angusitoflia* and *F. sonorensis* were calculated as well (Table 2).

### CS-based phylogeny construction of 16 Flaveria species

CS data matrix of 16 *Flaveria* species (excluding *F. pringlei*) using individual RNA-Seq library based on *m-CDS* (Fig. 3) and using pooled RNA-Seq from different library based on *m-CDS* (Fig. 4) and *s-CDS* (Additional file 4) were constructed. Data matrix of 16 *Flaveria* species (excluding *F. pringlei*) based on 1^st^, 2^nd^, and 3^rd^ codon positions were extracted from *m*-*CDS* using in-house Perl script (Additional file 5 A-C). We used PartitionFinder (V1.1.1) [57] to estimate the proper model for inferring phylogeny based on obtained CS data matrixes. The result showed GTR + GAMMA + I model best fitted these datasets. The same methods were then applied to infer the phylogeny based on these dataset respectively as mentioned above.

#### Accession number

The RNA-Seq datasets from 1KP Consortium were available at National Center for Biotechnology Information (NCBI) Gene Expression Omnibus (GEO) under accession number GSE54339. RNA-Seq data from HHU were available at NCBI Short Read Archive with accession number: SRX794138 for *F. angustifolia*, SRS777658 for *F. floridana*, SRS777663 for *F. vaginata,* SRS777671 for *F. palmeri*, SRS777680 for *F. australasica*, SRX468650 for *F. robusta* leaf, SRX794075 for *F. robusta* root, SRX794076 for *F. robusta* shoot, SRX468638 for *F. pubescens*, RX467630 for *F. chloreafolia*, SRX467620 for *F. anomala*, SRX467625 for *F. brownii*, SRX467614 for *F. bidentis* leaf, SRX794053 for *F. bidentis* root, SRX794064 for F. bidentis shoot, and SRX468662 for *F. trinervia*.

## Additional files

Additional file 1:
**The consistency of consensus sequence between mature leaf sample and juvenile leaf sample from the same species.** The X-axis shows the number of consensus sequence (CS) for each gene in mature leaf sample (MS), Y-axis is the number of identical CS between MS and juvenile leaf sample (JS). At median, 99.96 % sites were identical between MS and JS (Abbreviations: CS: consensus sequence, MS: mature leaf sample, JS: juvenile leaf sample). Additional file 2:
**Phylogenetic tree based on individual** ***Flaveria*** **sample based on** ***m-CDS.*** The *m-CDS* of *A. thaliana* that comprised only singleton genes was used as mapping reference to construct consensus sequence (CS) matrix according to Fig. 1. A CS matrix with 315,342 sites from 2,183 genes was used to infer phylogenetic relationships based on both Bayesian inference (BI) and Maximum likelihood (ML) using GTR + GAMMA + I model of sequence substitution and variation. BI tree and ML tree showed consistent topology. The numbers besides each node were posterior probability inferred from 1000,000 generations (up) and bootstrap score (down) from 500 bootstrap sampling (^#^/shoot^#^/root^#^/: leaf/shoot/root sample from HHU, j/m: juvenile/mature leaf sample from 1KP. *m-CDS*: reference contains the longest gene for each paralog family). Additional file 3:
**Estimation of the possibility of** ***F. pringlei*** **from HHU being a hybrid.** A shows four types of sites in *F. pringlei*: 1) hybrid type, if > = 40 % of mapping reads is same as the C_3_ marker and > = 40 % of mapping reads is same as C_3_-C_4_ marker. 2) C_3_-C_4_ type: if > = 90 % of mapping reads is the same as C_3_-C_4_ marker. 3) C_3_ type: if > = 90 % of mapping reads is the same as the C_3_ marker, and 4) others. B shows the proportion of four types. *F. pringlei* had about 70 % of sites being the hybrid type. C shows the proportion of C_3_ marker and C_3_-C_4_ marker of each hybrid site in *F. pringlei*. The definition of C_3_ marker and C_3_-C_4_ marker were defined in Methods. Additional file 4:
**Phylogeny of 16** ***Flaveria*** **species pooling data based on** ***s*** **-** ***CDS.*** Pooling samples of the same species from different developmental stages resulted in samples representing 16 *Flaveria* species. The *s*-*CDS* of *A. thaliana* was used as mapping reference to construct consensus sequence matrix. Both Bayesian inference (BI) tree and Maximum likelihood (ML) tree were inferred from 1,706 genes with 311,901 sites with GTR + GAMMA + I model of sequence substitution and variation. The numbers besides each node were posterior probability (up) inferred from 1000,000 generations and bootstrap score (down) from 500 bootstrap sampling. The numbers in brackets were relative branch length estimated from Bayesian (*s*-*CDS*: reference contains only singleton genes). Additional file 5:
**Phylogenetic tree of 16** ***Flaveria*** **species using three independent codon positions from** ***m-CDS.*** A: Phylogenetic tree based on 1^th^ codon sites. B: phylogenetic tree based on 2^nd^ codon sites. C: phylogenetic tree based on 3^rd^ codon sites. Pooling samples from different leaf libraries of one species resulted in samples representing 16 *Flaveria* species. Both Bayesian inference (BI) trees and Maximum likelihood (ML) tree were inferred from independent positions of codon from 2,271 genes with 191,482 sites using GTR+ GAMMA + I model of sequence substitution and variation. The numbers besides each node were posterior probability (up) inferred from 1000,000 generations and bootstrap score (down) from 500 bootstrap sampling (*m-CDS*: reference contains the longest gene for each paralog family).

## Abbreviations

mya: Million years ago
*m-CDS*: Minimal reference coding sequences
*s-CDS*: Singleton reference coding sequences
CS: Consensus sites
AS: Ambiguous sites
UNS: Uncovered site
M: Mature
J: Juvenile
ML: Maximum likelihood
BI: Bayesian inference
