# Supplementary material for: RNA-Seq based phylogeny recapitulates previous phylogeny of the genus Flaveria (Asteraceae) with some modifications
Source: BMC Evol Biol. 2015 Jun 18;15:116. doi: 10.1186/s12862-015-0399-9 (PMC4472175; doi:10.1186/s12862-015-0399-9)
Supplement: Additional file 1: — The consistency of consensus sequence between mature leaf sample and juvenile leaf sample from the same species. The X-axis shows the number of consensus sequence (CS) for each gene in mature leaf sample (MS), Y-axis is the number of identical CS between MS and juvenile leaf sample (JS). At median, 99.96 % sites were identical between MS and JS (Abbreviations: CS: consensus sequence, MS: mature leaf sample, JS: juvenile leaf sample). [file 12862_2015_399_MOESM1_ESM.doc]

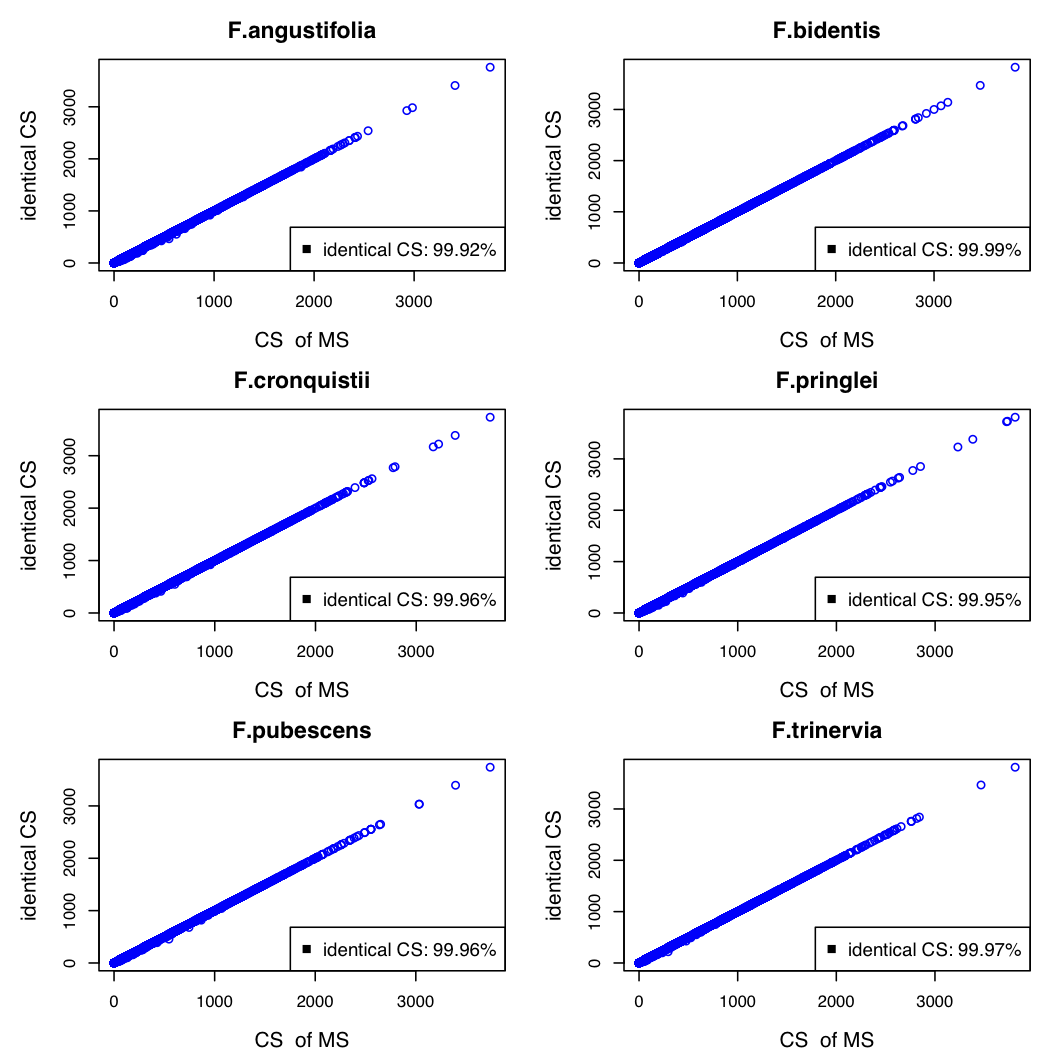


Additional file 1: The consistency of consensus sequence between mature leaf sample and juvenile leaf sample from the same species.

The X-axis shows the number of consensus sequence (CS) for each gene in mature leaf sample (MS), Y-axis is the number of identical CS between MS and juvenile leaf sample (JS). At median, 99.96% sites were identical between MS and JS. (Abbreviations: CS: consensus sequence, MS: mature leaf sample, JS: juvenile leaf sample.)
