# Supplementary material for: RNA-Seq based phylogeny recapitulates previous phylogeny of the genus Flaveria (Asteraceae) with some modifications
Source: BMC Evol Biol. 2015 Jun 18;15:116. doi: 10.1186/s12862-015-0399-9 (PMC4472175; doi:10.1186/s12862-015-0399-9)
Supplement: Additional file 2: — Phylogenetic tree based on individual Flaveria sample based on m-CDS. The m-CDS of A. thaliana that comprised only singleton genes was used as mapping reference to construct consensus sequence (CS) matrix according to Fig. 1. A CS matrix with 315,342 sites from 2,183 genes was used to infer phylogenetic relationships based on both Bayesian inference (BI) and Maximum likelihood (ML) using GTR + GAMMA + I model of sequence substitution and variation. BI tree and ML tree showed consistent topology. The numbers besides each node were posterior probability inferred from 1000,000 generations (up) and bootstrap score (down) from 500 bootstrap sampling (#/shoot#/root#/: leaf/shoot/root sample from HHU, j/m: juvenile/mature leaf sample from 1KP. m-CDS: reference contains the longest gene for each paralog family). [file 12862_2015_399_MOESM2_ESM.doc]

Additional file 2：Phylogenetic tree of individual *Flaveria* sample based on *m-*CDS

The *m*-CDS of *A. thaliana* that comprised only singleton genes was used as mapping reference to construct consensus sequence (CS) matrix according to Figure 1. A CS matrix with 315,342 sites from 2,183 genes was used to infer phylogenetic relationships based on both Bayesian and ML using GTR+GAMMA+I model of sequence substitution and variation. Bayesian tree and ML tree showed consistent topology. The numbers besides each node were posterior probability inferred from 1000,000 generations (up) and bootstrap score (down) from 500 bootstrap sampling. (#/shoot^#^/root^#^/: leaf/shoot/root sample from HHU, j/m: juvenile/mature leaf sample from 1KP. *m-CDS*: reference contains the longest gene for each paralog family*.*)
