# Supplementary material for: RNA-Seq based phylogeny recapitulates previous phylogeny of the genus Flaveria (Asteraceae) with some modifications
Source: BMC Evol Biol. 2015 Jun 18;15:116. doi: 10.1186/s12862-015-0399-9 (PMC4472175; doi:10.1186/s12862-015-0399-9)
Supplement: Additional file 3: — Estimation of the possibility of F. pringlei from HHU being a hybrid. A shows four types of sites in F. pringlei: 1) hybrid type, if > = 40 % of mapping reads is same as the C3 marker and > = 40 % of mapping reads is same as C3-C4 marker. 2) C3-C4 type: if > = 90 % of mapping reads is the same as C3-C4 marker. 3) C3 type: if > = 90 % of mapping reads is the same as the C3 marker, and 4) others. B shows the proportion of four types. F. pringlei had about 70 % of sites being the hybrid type. C shows the proportion of C3 marker and C3-C4 marker of each hybrid site in F. pringlei. The definition of C3 marker and C3-C4 marker were defined in Methods. [file 12862_2015_399_MOESM3_ESM.doc]

Additional file 3: Estimation of the possibility of *F. pringlei* from HHU being a hybrid

A shows four types of sites in *F. pringlei*: 1) hybrid type, if >=40% of mapping reads is same as the C_3_ marker and >=40% of mapping reads is same as C_3_-C_4_ marker. 2) C_3_-C_4_ type: if >=90% of mapping reads is the same as C_3_-C_4_ marker. 3) C_3_ type: if >=90% of mapping reads is the same as the C_3_ marker, and 4) others. B shows the proportion of four types. *F. pringlei* had about 70% of sites being the hybrid type. C shows the proportion of C_3_ marker and C_3_-C_4_ marker of each hybrid site in *F. pringlei*. The definition of C_3_ marker and C_3_-C_4_ marker were defined in Methods.
