# Supplementary material for: RNA-Seq based phylogeny recapitulates previous phylogeny of the genus Flaveria (Asteraceae) with some modifications
Source: BMC Evol Biol. 2015 Jun 18;15:116. doi: 10.1186/s12862-015-0399-9 (PMC4472175; doi:10.1186/s12862-015-0399-9)
Supplement: Additional file 4: — Phylogeny of 16 Flaveria species pooling data based on s - CDS. Pooling samples of the same species from different developmental stages resulted in samples representing 16 Flaveria species. The s-CDS of A. thaliana was used as mapping reference to construct consensus sequence matrix. Both Bayesian inference (BI) tree and Maximum likelihood (ML) tree were inferred from 1,706 genes with 311,901 sites with GTR + GAMMA + I model of sequence substitution and variation. The numbers besides each node were posterior probability (up) inferred from 1000,000 generations and bootstrap score (down) from 500 bootstrap sampling. The numbers in brackets were relative branch length estimated from Bayesian (s-CDS: reference contains only singleton genes). [file 12862_2015_399_MOESM4_ESM.doc]

Additional file 4: Phylogeny of 16 *Flaveria* species pooling data based on *s*-*CDS*

Pooling samples of the same species from different developmental stages resulted in samples representing 16 *Flaveria* species. The *s*-*CDS* of *A. thaliana* was used as mapping reference to construct consensus sequence matrix. Both Bayesian inference (BI) tree and Maximum likelihood (ML) tree were inferred from 1,706 genes with 311,901 sites with GTR+GAMMA+I model of sequence substitution and variation. The numbers besides each node were posterior probability (up) inferred from 1000,000 generations and bootstrap score (down) from 500 bootstrap sampling. The numbers in brackets were relative branch length estimated from Bayesian. (*s*-*CDS*: reference contains only singleton genes.)
