# Supplementary material for: RNA-Seq based phylogeny recapitulates previous phylogeny of the genus Flaveria (Asteraceae) with some modifications
Source: BMC Evol Biol. 2015 Jun 18;15:116. doi: 10.1186/s12862-015-0399-9 (PMC4472175; doi:10.1186/s12862-015-0399-9)
Supplement: Additional file 5: — Phylogenetic tree of 16 Flaveria species using three independent codon positions from m-CDS. A: Phylogenetic tree based on 1th codon sites. B: phylogenetic tree based on 2nd codon sites. C: phylogenetic tree based on 3rd codon sites. Pooling samples from different leaf libraries of one species resulted in samples representing 16 Flaveria species. Both Bayesian inference (BI) trees and Maximum likelihood (ML) tree were inferred from independent positions of codon from 2,271 genes with 191,482 sites using GTR+ GAMMA + I model of sequence substitution and variation. The numbers besides each node were posterior probability (up) inferred from 1000,000 generations and bootstrap score (down) from 500 bootstrap sampling (m-CDS: reference contains the longest gene for each paralog family). [file 12862_2015_399_MOESM5_ESM.doc]

Additional file 5: Phylogenetic tree of 16 *Flaveria* species using three independent codon positions from *m-CDS*

A: Phylogenetic tree based on 1^th^ codon sites. B: phylogenetic tree based on 2^nd^ codon sites. C: phylogenetic tree based on 3^rd^ codon sites. Pooling samples from different leaves libraries of one species resulted in samples representing 16 *Flaveria* species. Both Bayesian inference (BI) and Maximum likelihood (ML) tree were inferred from independent positions of codon from 2,271 genes with 191,482 sites using GTR+ GAMMA+I model of sequence substitution and variation. The numbers besides each node were posterior probability (up) inferred from 1000,000 generations and bootstrap score (down) from 500 bootstrap sampling. (*m-CDS*: reference contains the longest gene for each paralog family).
